# Supplementary material for: Protein design using structure-based residue preferences
Source: Nat Commun. 2024 Feb 22;15:1639. doi: 10.1038/s41467-024-45621-4 (PMC10884402; doi:10.1038/s41467-024-45621-4)
Supplement: Supplementary file 1 — Supplementary Information [file 41467_2024_45621_MOESM1_ESM.pdf]

Supplementary Information

for

"Protein design using structure-based residue preferences"

Authors: David Ding[1*], Ada Shaw[2], Sam Sinai[3], Nathan Rollins[4], Noam Prywes[1], David F. Savage[1,5,6], Michael T. Laub[7,8], Debora S. Marks[2*]

Affiliations:

[1] Innovative Genomics Institute, University of California; Berkeley, CA 94720, USA.

[2] Department of Systems Biology, Harvard Medical School; Boston, MA, 02115, USA.

[3] Dyno Therapeutics, Watertown, MA, 02472, USA.

[4] Seismic Therapeutics, Lab Central; Cambridge, MA, 02142, USA.

[5] Department of Molecular and Cell Biology, University of California; Berkeley, CA, 94720, USA.

[6] Howard Hughes Medical Institute, University of California; Berkeley, CA, 94720, USA.

[7] Department of Biology, Massachusetts Institute of Technology; Cambridge, MA 02139, USA.

[8] Howard Hughes Medical Institute, Massachusetts Institute of Technology; Cambridge, MA 02139, USA.

*Corresponding authors: debbie@hms.harvard.edu, davidding@berkeley.edu

Supplementary Figures 1-9

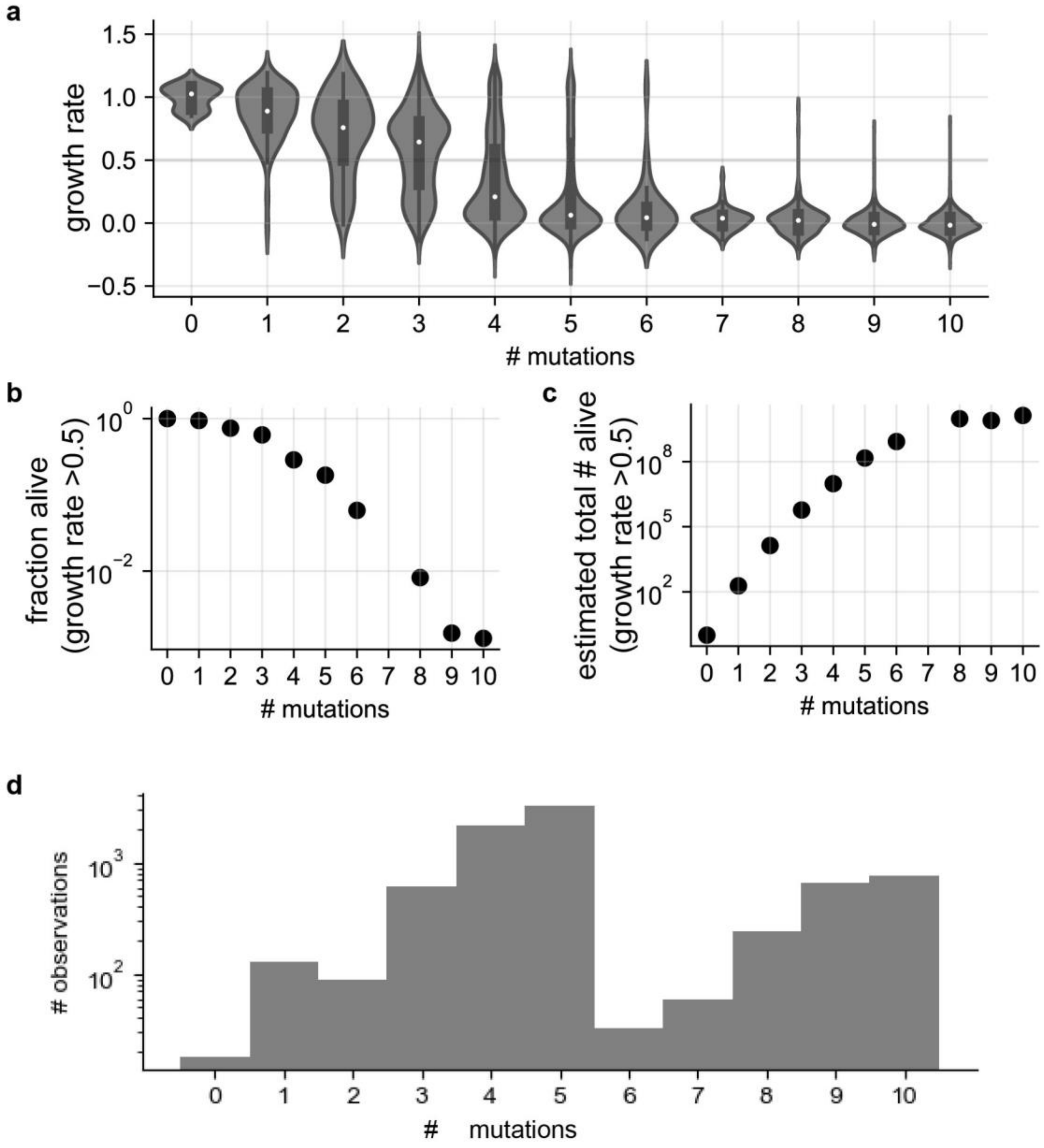

Supplementary Figure 1: Observed statistics of 10 position antitoxin ParD3 library enable predicting the total number of functional antitoxin variants.
a, The distribution of antitoxin variant growth rate effects split across the number of substitutions.
b-c, The fraction of antitoxin variants that reach half-maximal growth rate values as a function of substitution distance (**b**), and the resulting estimated total number of antitoxin variants that reach half-maximal neutralization at a given substitution distance (**c**).
d, The number of observations for each number of substitutions of antitoxin variants is shown.

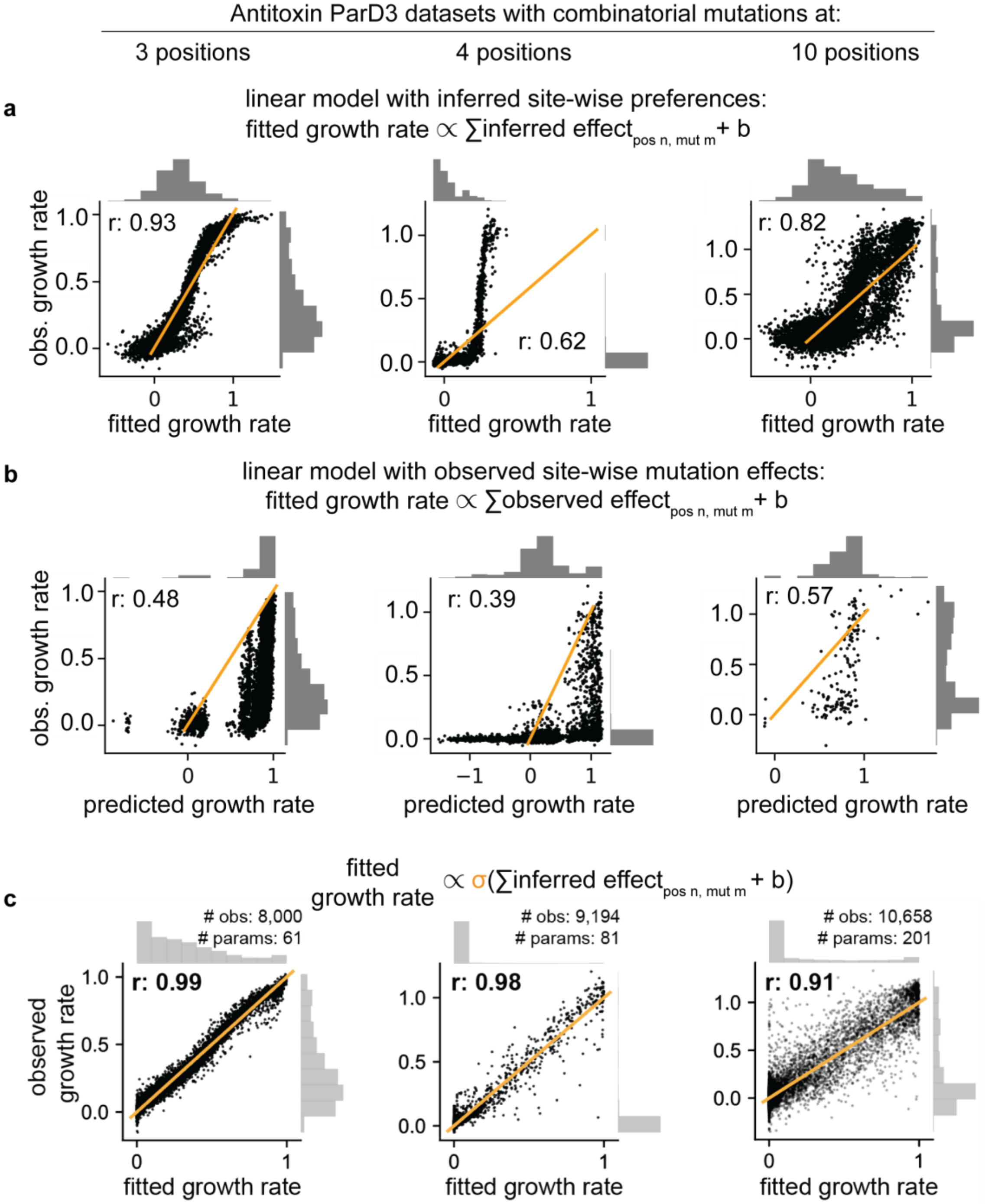

Supplementary Figure 2: Fit of linear site-wise preference model and observed site-wise mutation effects.

a, Fit of a linear model using inferred site-wise preferences as predictors, without a sigmoid nonlinearity, for observed combinatorial variant effects.

b, Fit of a linear model using observed single variant effects as predictors for observed combinatorial variant effects.

c, Fit of a logistic model using inferred single variant effects as predictors for observed combinatorial variant effects. This corresponds to Fig. 2g.

Pearson correlation coefficients (r) are indicated.

22

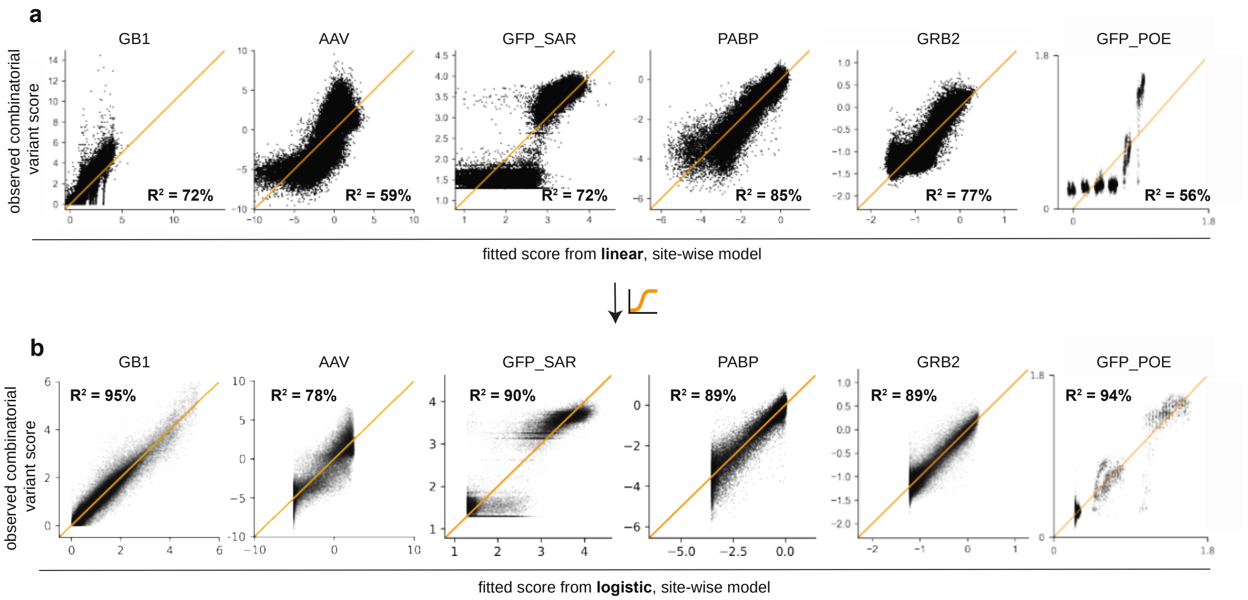

Supplementary Figure 3: Logistic regression with site-wise mutation preferences enables better fit to combinatorial variant effect datasets across proteins.
a-b, Site-wise linear regression (**a**) and logistic regression (**b**) fits to the total combinatorial variant effects for various proteins, with the total explained variance R^2 indicated.

23

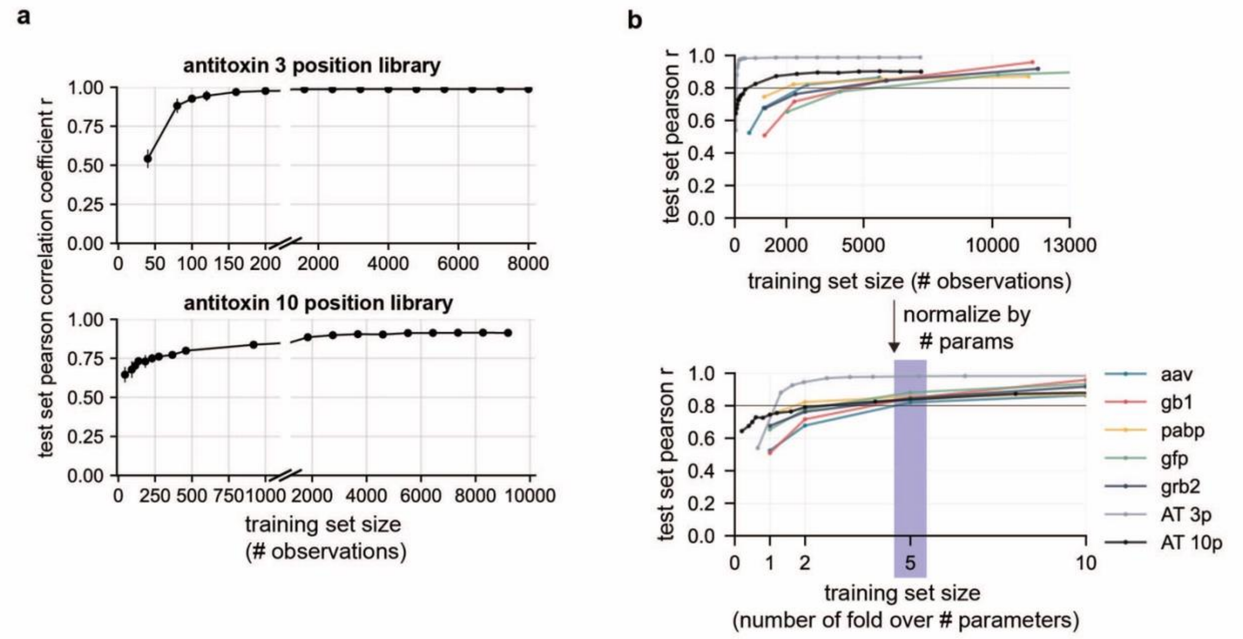

Supplementary Figure 4: Few observed sequences are required to predict combinatorial mutation effects with a logistic model using residue mutation preferences.

a,The nonlinear, additive model was trained on a subset of combinatorial variants, and then used to predict the remaining held-out combinatorial mutation effects for the 3 position (top) or 10 position antitioxin library (bottom). The pearson correlation coefficient, r, between predicted and observed variant effects is indicated vs. the number of subsampled observations used to infer the site-wise amino acid preferences. Errorbars represent standard deviations from repeated random subsampling (n=8).

b, A subset of the observed combinatorial variant effects were used to fit the site-wise preference parameters of a logistic model for 6 proteins and their performance on the remaining held-out combinatorial variant effects in each dataset is shown. The top graph shows the absolute number of training examples used, while the bottom graph shows the number of training examples used to fit the model in terms of fold over the number of site-wise preference parameters used by the model depending on the number of mutated residues in each dataset.

24

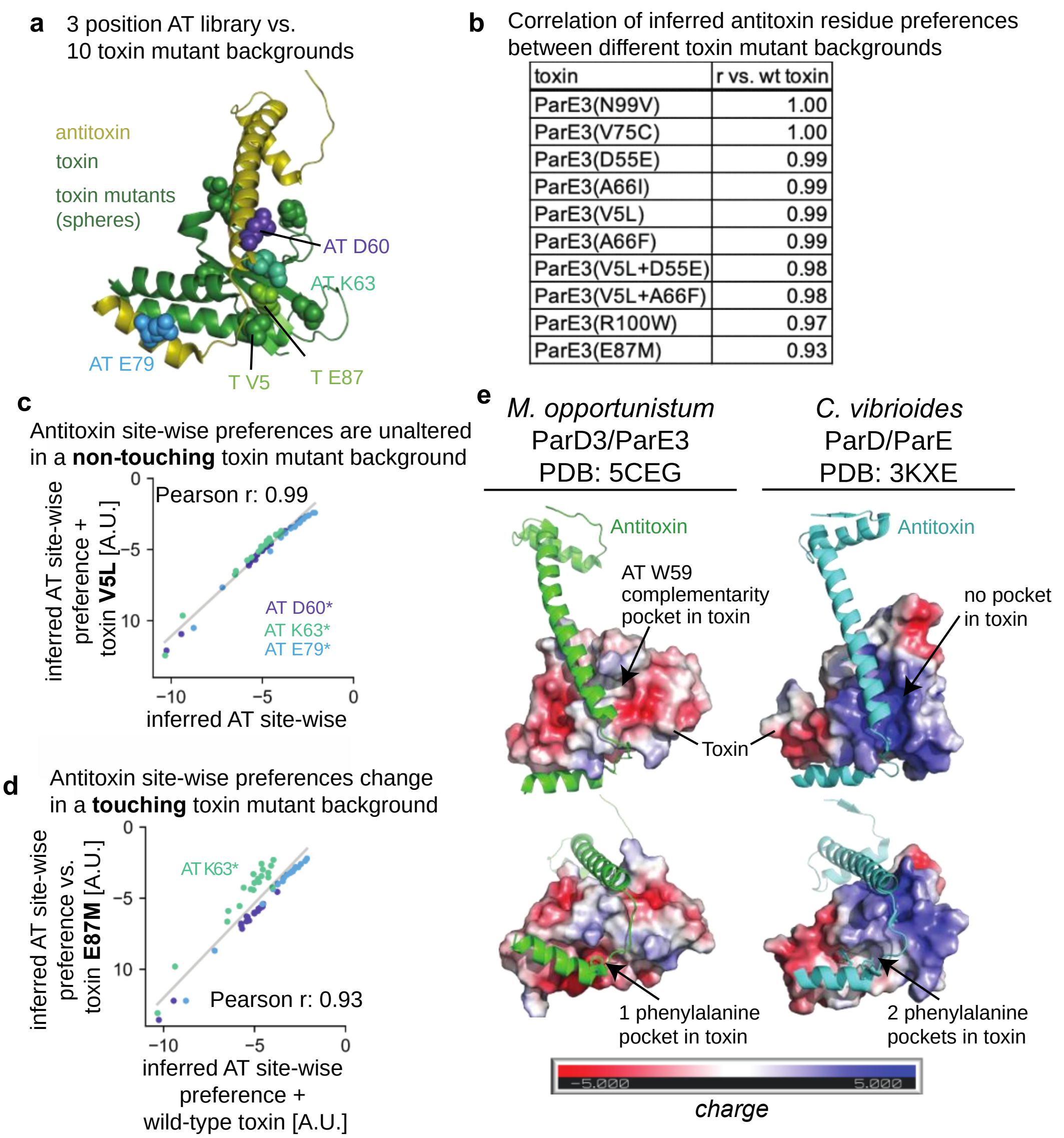

toxin	r vs. wt toxin
ParE3(N99V)	1.00
ParE3(V75C)	1.00
ParE3(D55E)	0.99
ParE3(A66I)	0.99
ParE3(V5L)	0.99
ParE3(A66F)	0.99
ParE3(V5L+D55E)	0.98
ParE3(V5L+A66F)	0.98
ParE3(R100W)	0.97
ParE3(E87M)	0.93

Supplementary Figure 5: Antitoxin site-wise preferences are altered by mutations in a contacting residue, and homologous structure comparison suggest differences in site-wise preferences between homologous toxin-antitoxin pairs.

a, Crystal structure (PDB ID: 5CEG) indicating the 10 different ParE3 toxin single substitution variants that the 3 position combinatorial antitoxin ParD3 library was screened against. The toxin is shown in green, with the mutated positions spacefilled. The antitoxin is shown in yellow with purple, cyan and blue indicating the combinatorially mutated residues. Only one mutated position in the toxin, E87, contacts the antitoxin at position K63 (cyan).

b-d,Antitoxin ParD3 site-wise preferences inferred by the nonlinear, independent model correlate almost perfectly unless a contacting residue is mutated. **b,** Pearson correlation coefficients, r, for inferred sitewise antitoxin preferences at each of the 3 mutated antitoxin positions in the background of 10 different toxin ParE3 single substitution variants vs. the wild-type toxin ParE3. Scatterplot of antitoxin residue mutation preferences when screened against toxin with a non-contacting mutation (V5L) vs. wild-type toxin (**c**). Differences in antitoxin position K63 mutation preferences in the background of a toxin variant that contains a substitution, E87M, at a contacting position (**d**).

e, Differences in local interface microenvironments of binding between ParD3/E3 from *Mesorhizobium opportunistum* (PDB ID: 5CEG) vs. homologous structure ParD/E (PDB ID: 3KXE) from *Caulobacter vibrioides*, suggesting that alignment-averaged amino acid preferences do not reflect the site-wise preference of each individual structural context well.

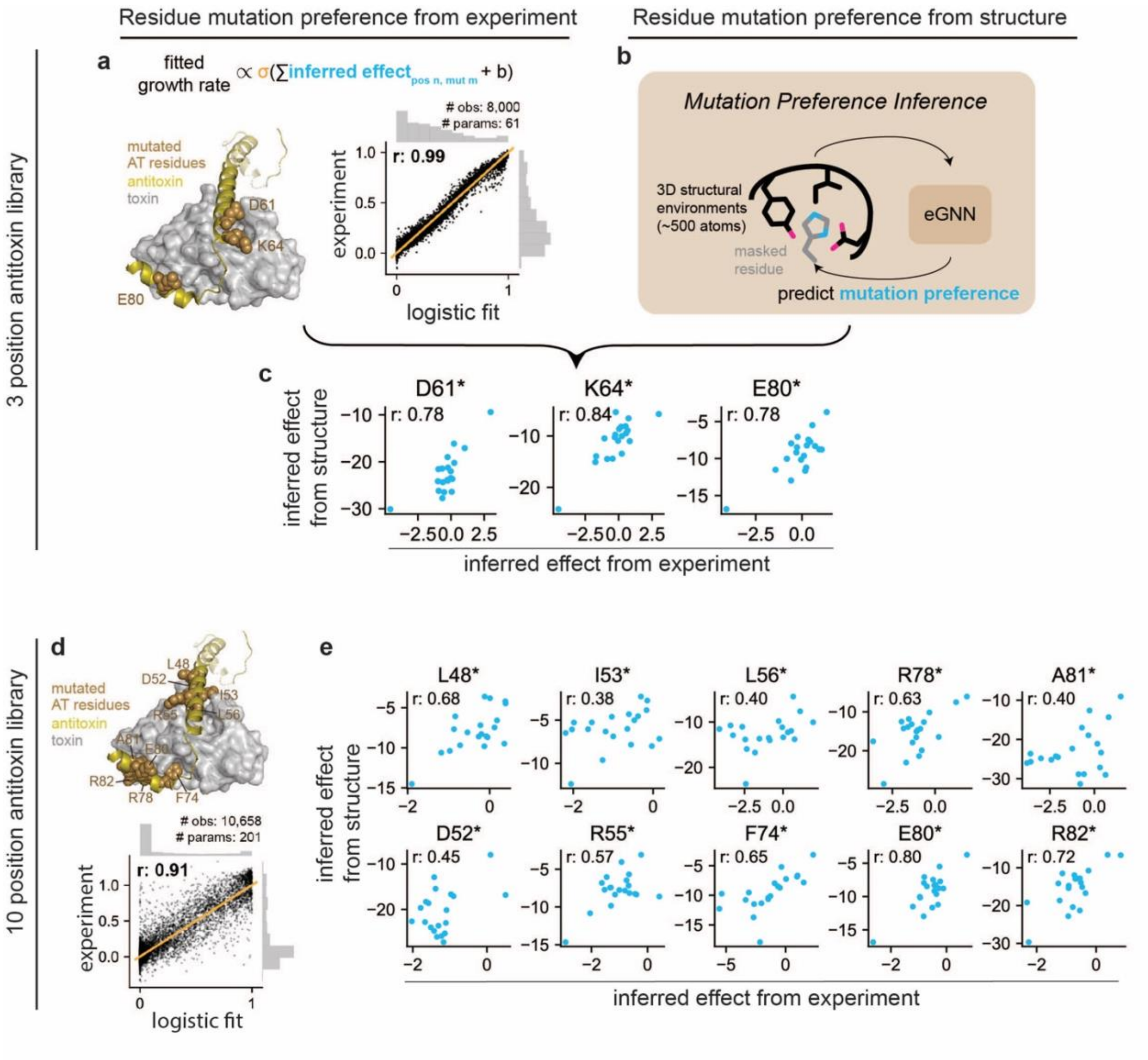

Supplementary Figure 6: Antitoxin residue mutation preferences can be learned from structural context.
a, 60 residue mutation preferences at three antitoxin positions learnd from the experiment can explain almost all of the combinatorial variant effects.
b, Mutation preferences can be learned from structural context without variant effect measurements.
c, Correlation between experimental and unsupervised, structure-based inference of mutation preferences.
d-e, Per residue mutation preferences can be learned from structural context for the 10 position antitoxin library, with the explanatory power of the inferred residue preferences shown (**d**).
Pearson correlation coefficients r indicated.

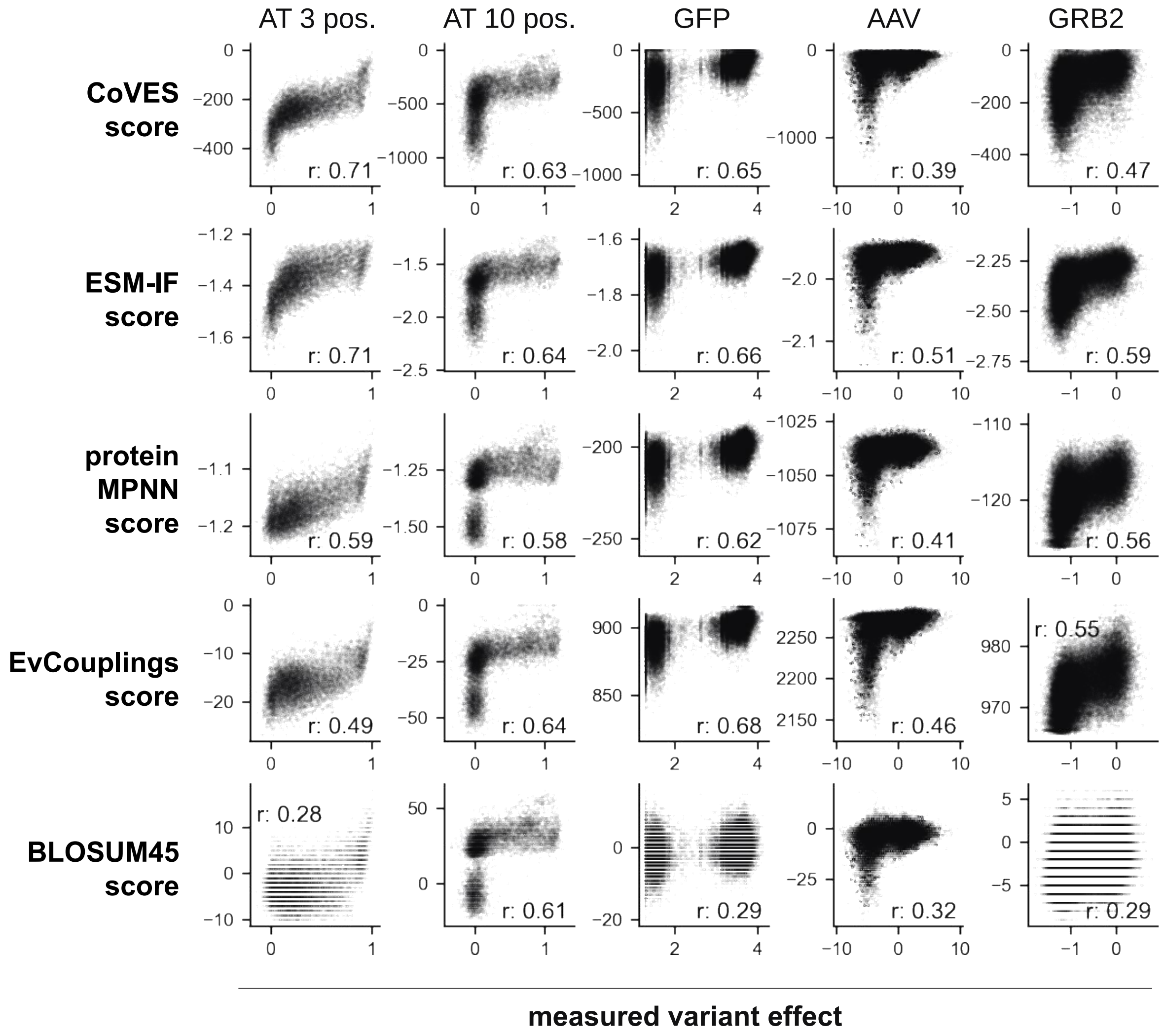

Supplementary Figure 7: Unsupervised model scores correlate with measured combinatorial variant effects.
The scores for various models (CoVES, ESM-IF, proteinMPNN, EvCouplings and BLOSUM45) are displayed versus the measured variant effects for the antitoxin ParD3 3 position library, antitoxin 10 position library, GFP, AAV and GRB2 datasets. The spearman correlation coefficients (r) are indicated.

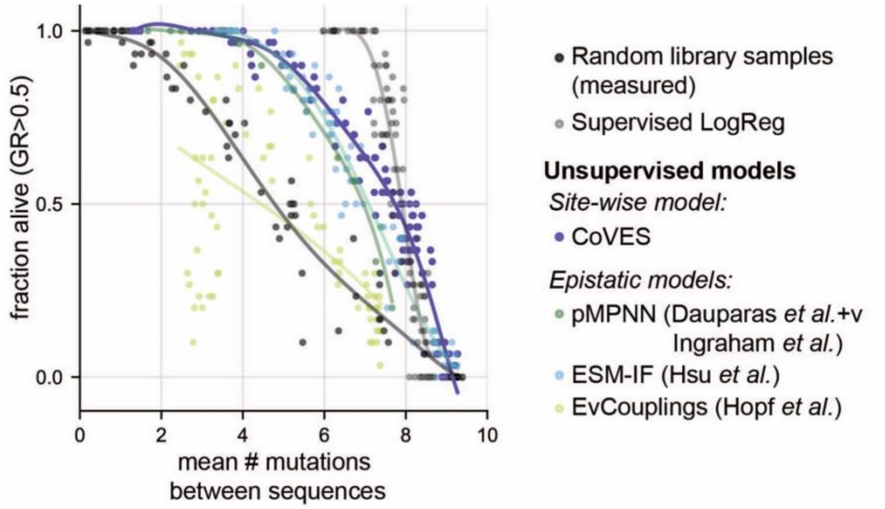

Supplementary Figure 8: Designed antitoxin sequences at 10 positions from unsupervised models evaluated for function and between sequence diversity.
Scatterplot showing the fraction of designed sequences that are predicted to be functional and the average number of mutations between designed sequences. Each dot represents 30 unique sequences sampled by each supervised model at a given sampling temperature.

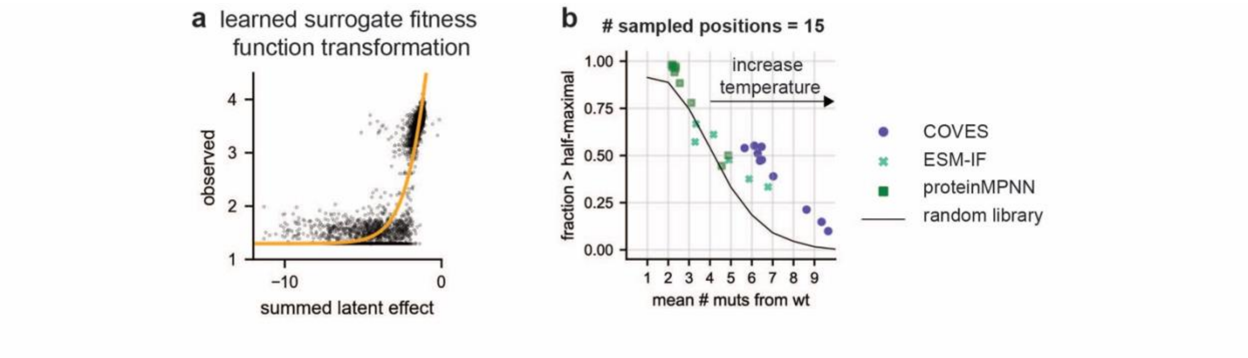

Supplementary Figure 9: Surrogate fitness function evaluation of designed GFP variants from unsupervised sequence models.
a, The learned nonlinear transformation of the supervised surrogate fitness function using the summed inferred residue-wise mutation preferences to predict observed combinatorial variant effects is shown.
b, The diversity and function of sampled sequences from unsupervised models is shown. Each dot represents a set of sampled sequences at a given temperature and number of positions that are randomized, summarized by the fraction of sequences predicted to be above half-maximal fluorescence and the average number of mutations. The maximum number of mutated positions is set to 15. Only combinatorial variants consisting of individual variants, which the oracle logistic regression has been trained on and tested are shown.
