# Supplementary material for: Protein design using structure-based residue preferences
Source: Nat Commun. 2024 Feb 22;15:1639. doi: 10.1038/s41467-024-45621-4 (PMC10884402; doi:10.1038/s41467-024-45621-4)
Supplement: Supplementary file 3 — Description of Additional Supplementary Files [file 41467_2024_45621_MOESM3_ESM.pdf]

Description of Additional Supplementary Files:

Supplementary Data 1: Primers used in this study.

Supplementary Data 2: Sequence fragments of antitoxin variants designed by CoVES.
