# Supplementary material for: Protein design using structure-based residue preferences
Source: Nat Commun. 2024 Feb 22;15:1639. doi: 10.1038/s41467-024-45621-4 (PMC10884402; doi:10.1038/s41467-024-45621-4)
Supplement: Supplementary file 6 — Reporting Summary [file 41467_2024_45621_MOESM6_ESM.pdf]

nature portfolio

Corresponding author(s): Debora S Marks, David Ding

Last updated by author(s): Dec 18, 2023

Reporting Summary

Nature Portfolio wishes to improve the reproducibility of the work that we publish. This form provides structure for consistency and transparency in reporting. For further information on Nature Portfolio policies, see our Editorial Policies and the Editorial Policy Checklist.

Statistics

For all statistical analyses, confirm that the following items are present in the figure legend, table legend, main text, or Methods section.

n/a	Confirmed	
☐	☒	The exact sample size (n) for each experimental group/condition, given as a discrete number and unit of measurement
☐	☒	A statement on whether measurements were taken from distinct samples or whether the same sample was measured repeatedly
☒	☐	The statistical test(s) used AND whether they are one- or two-sided<br>*Only common tests should be described solely by name; describe more complex techniques in the Methods section.*
☐	☒	A description of all covariates tested
☐	☒	A description of any assumptions or corrections, such as tests of normality and adjustment for multiple comparisons
☐	☒	A full description of the statistical parameters including central tendency (e.g. means) or other basic estimates (e.g. regression coefficient) AND variation (e.g. standard deviation) or associated estimates of uncertainty (e.g. confidence intervals)
☒	☐	For null hypothesis testing, the test statistic (e.g. F, t, r) with confidence intervals, effect sizes, degrees of freedom and P value noted<br>*Give P values as exact values whenever suitable.*
☒	☐	For Bayesian analysis, information on the choice of priors and Markov chain Monte Carlo settings
☒	☐	For hierarchical and complex designs, identification of the appropriate level for tests and full reporting of outcomes
☐	☒	Estimates of effect sizes (e.g. Cohen's d, Pearson's r), indicating how they were calculated

Our web collection on statistics for biologists contains articles on many of the points above.

Software and code

Policy information about availability of computer code

Data collection	FLASH 1.2.11, vsearch 2.13.0
Data analysis	Tensorflow 2, custom code available at: https://github.com/ddingding/CoVES

For manuscripts utilizing custom algorithms or software that are central to the research but not yet described in published literature, software must be made available to editors and reviewers. We strongly encourage code deposition in a community repository (e.g. GitHub). See the Nature Portfolio guidelines for submitting code & software for further information.

Data

Policy information about availability of data

All manuscripts must include a data availability statement. This statement should provide the following information, where applicable:

- Accession codes, unique identifiers, or web links for publicly available datasets
- A description of any restrictions on data availability
- For clinical datasets or third party data, please ensure that the statement adheres to our policy

Raw sequencing read data is available at NCBI BioProject: PRJNA988522.
DMS data sources:
- antitoxin 3-position library: Ding et al., 2022.
- GB1: Olson et al., 2014.

- PABP: Melamed et al., 2013.
- GRB2: Faure et al., 2022.
- AAV: Bryant et al., 2021.
- GFP_SAR: Sarkisyan et al., 2016.
- GFP_POE: Poelwijk et al., 2019.

Used PDB IDs: 5CEG, 1LP3, 1PGA, 1FCC, 2WUR, 2VWF, 1CVJ.

Research involving human participants, their data, or biological material

Policy information about studies with human participants or human data. See also policy information about sex, gender (identity/presentation), and sexual orientation and race, ethnicity and racism.

Reporting on sex and gender	N/A
Reporting on race, ethnicity, or other socially relevant groupings	N/A
Population characteristics	N/A
Recruitment	N/A
Ethics oversight	N/A

Note that full information on the approval of the study protocol must also be provided in the manuscript.

Field-specific reporting

Please select the one below that is the best fit for your research. If you are not sure, read the appropriate sections before making your selection.

☐ Life sciences ☐ Behavioural & social sciences ☒ Ecological, evolutionary & environmental sciences

For a reference copy of the document with all sections, see nature.com/documents/nr-reporting-summary-flat.pdf

Ecological, evolutionary & environmental sciences study design

All studies must disclose on these points even when the disclosure is negative.

Study description	This study examines fits of different functions to combinatorial protein variant effect datasets, including one generated here. It also examines the inference of per residue mutation preferences from structural context and the sampling of combinatorial variants using these in comparison to other methods. We gathered combinatorial variant effect measurements in the antitoxin, where 2 biological replicates were measured. The experimental units consist of individual variants.
Research sample	The samples consist of variant effect datasets generate here and elsewhere. They arise due to the ability in each specific system to measure >1000s of protein variant effects in a single assay, and for containing combinations of variant effects. These samples were chosen for being combinatorial variant effect measurements, representing in certain instances a population of higher order combinatorial variant effects. The data sources are from prior published literature (see Data availability).
Sampling strategy	We performed experiments in replicate, and performed no sample size calculation. Replicates were chosen for experimental tractability. This sample size is sufficient to show high reproducibility in measuring variant effects.
Data collection	David Ding recorded the toxin-antitoxin dataset generated here, using high-throughput sequencing of variants as described previously. Samples were recorded by sampling 50ml samples from a 250 milliliter bulk bacterial growth population.
Timing and spatial scale	Samples were collected in December 2022. Sampling was performed at toxin expression induction in the bulk growth measurement assay, and 10 hours later, as performed previously. These samples were taken from bulk flask bacterial growth conditions with no spatial scale involved.
Data exclusions	Variant effects with less than 3 reads per variant were excluded in order to minimize low-read noise.
Reproducibility	The experiment was performed in biological replicate (separate transformations), and all attempts were successful.
Randomization	The library is fully randomized.
Blinding	In these experiments, the scientist is blinded to the variant effect measurements during the experiment and sampling, but the variants are during after high-throughput sequencing analysis. No other approach to blinding is possible.

Did the study involve field work? ☐ Yes ☒ No

Reporting for specific materials, systems and methods

We require information from authors about some types of materials, experimental systems and methods used in many studies. Here, indicate whether each material, system or method listed is relevant to your study. If you are not sure if a list item applies to your research, read the appropriate section before selecting a response.

Materials & experimental systems

n/a	Involved in the study
☒	☐ Antibodies
☒	☐ Eukaryotic cell lines
☒	☐ Palaeontology and archaeology
☒	☐ Animals and other organisms
☒	☐ Clinical data
☒	☐ Dual use research of concern
☒	☐ Plants

Methods

n/a	Involved in the study
☒	☐ ChIP-seq
☒	☐ Flow cytometry
☒	☐ MRI-based neuroimaging

Plants

Seed stocks	N/A
Novel plant genotypes	N/A
Authentication	N/A
