# Supplementary figures and images for: Protein design using structure-based residue preferences
Source: Nat Commun. 2024 Feb 22;15:1639. doi: 10.1038/s41467-024-45621-4 (PMC10884402; doi:10.1038/s41467-024-45621-4)

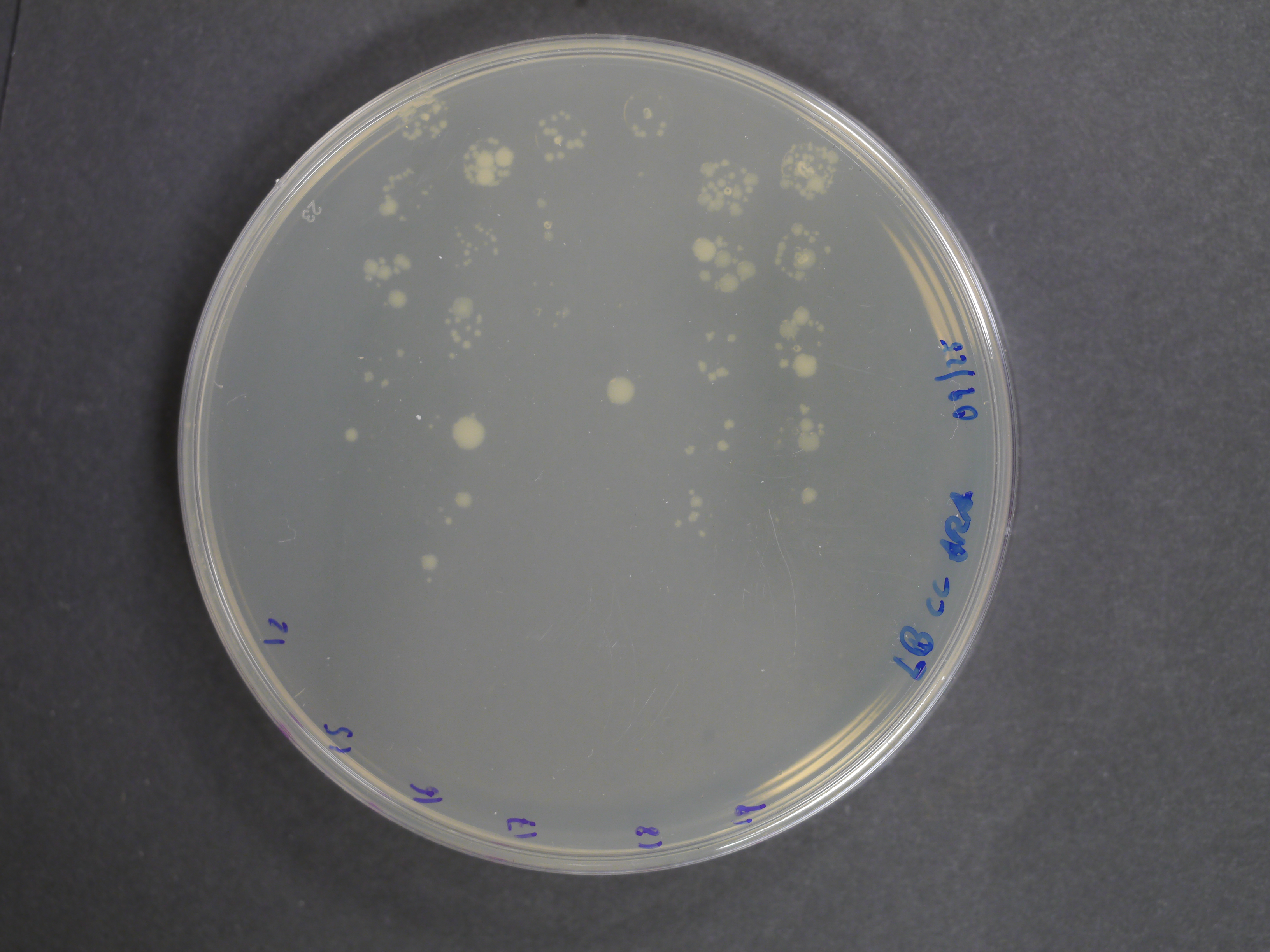

Supplement: Supplementary file 7 — Source Data [file 41467_2024_45621_MOESM7_ESM.zip › source/fig3c/P1160179.JPG]

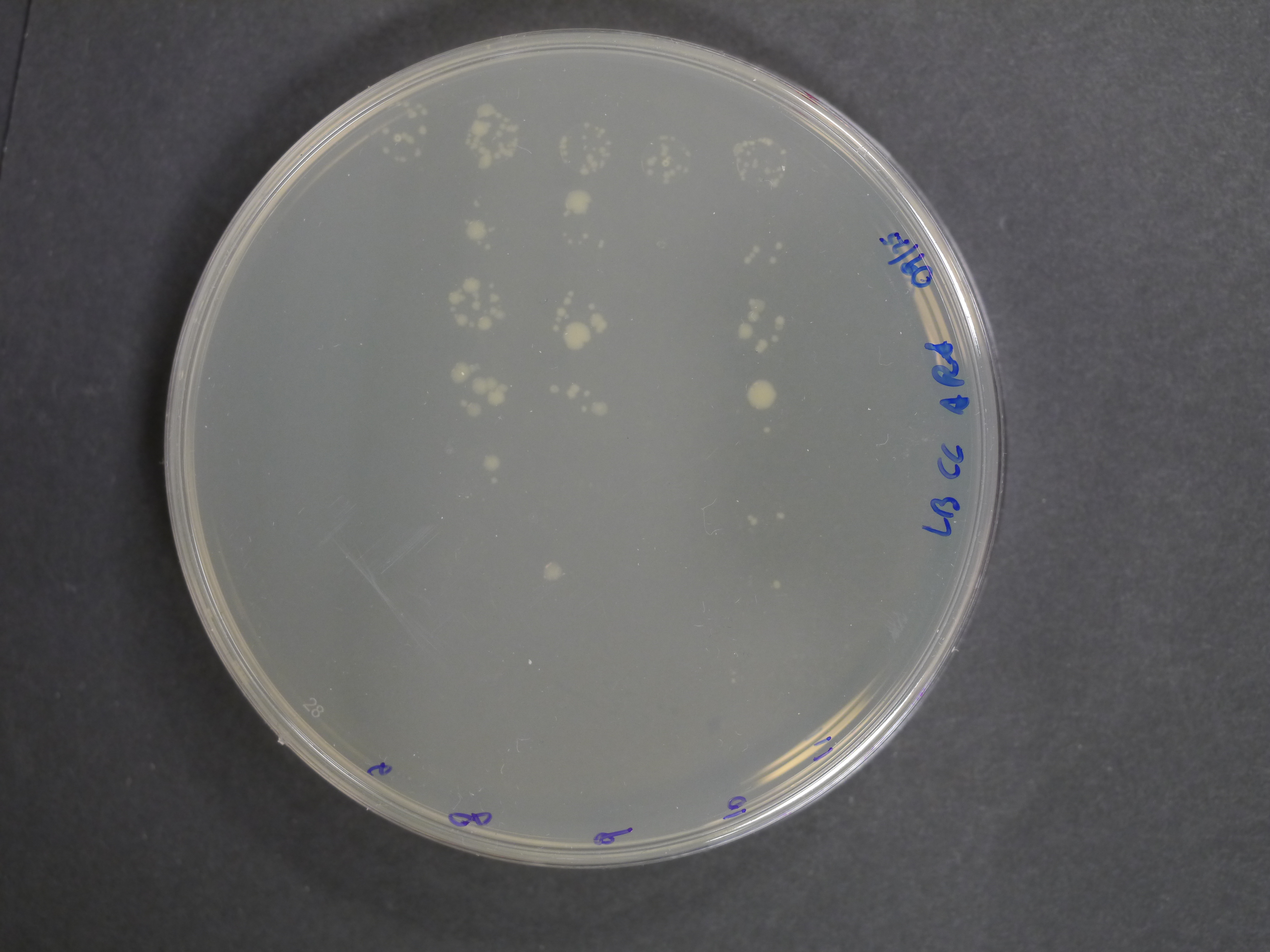

Supplement: Supplementary file 7 — Source Data [file 41467_2024_45621_MOESM7_ESM.zip › source/fig3c/P1160178.JPG]

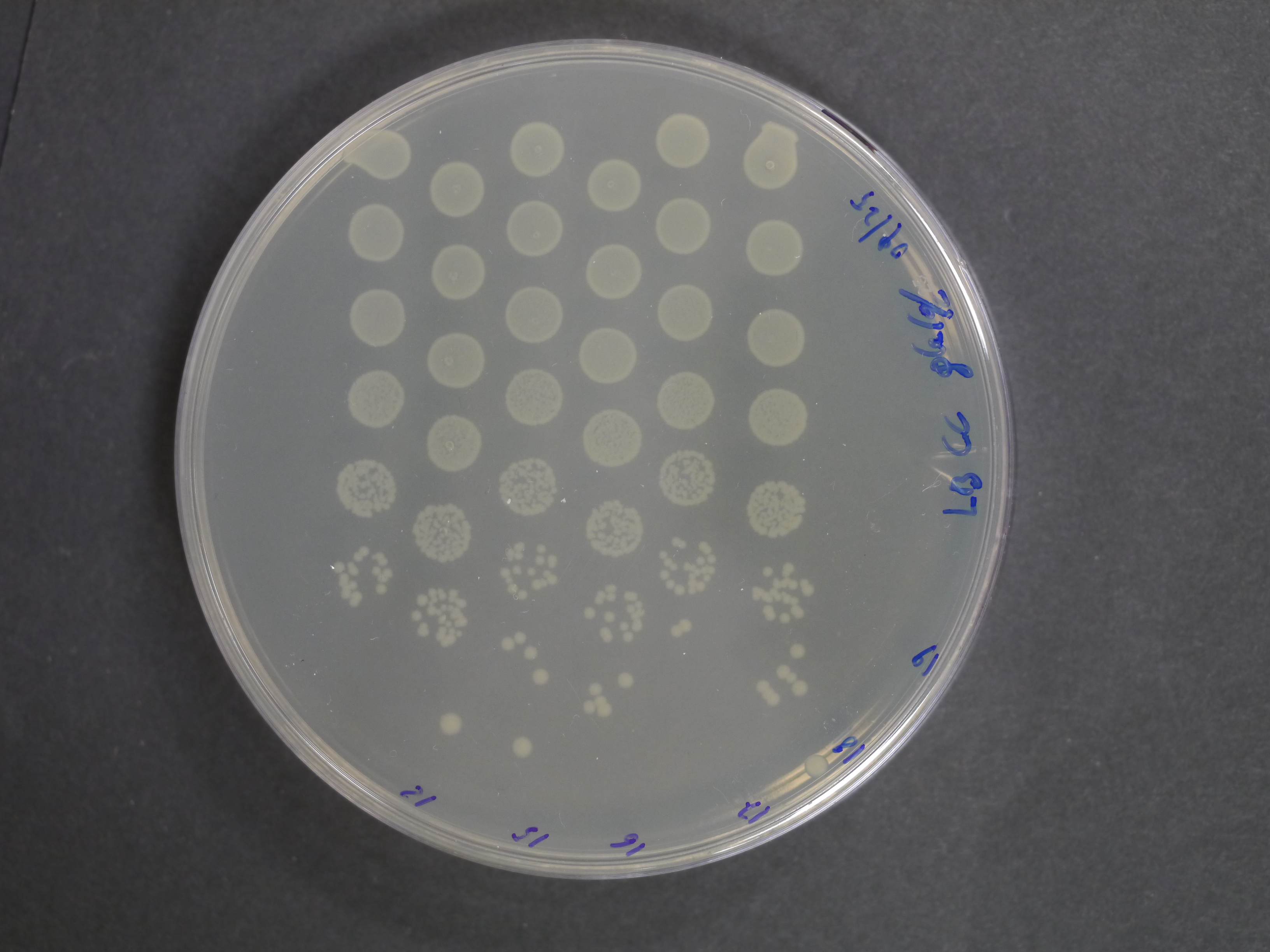

Supplement: Supplementary file 7 — Source Data [file 41467_2024_45621_MOESM7_ESM.zip › source/fig3c/P1160176.JPG]

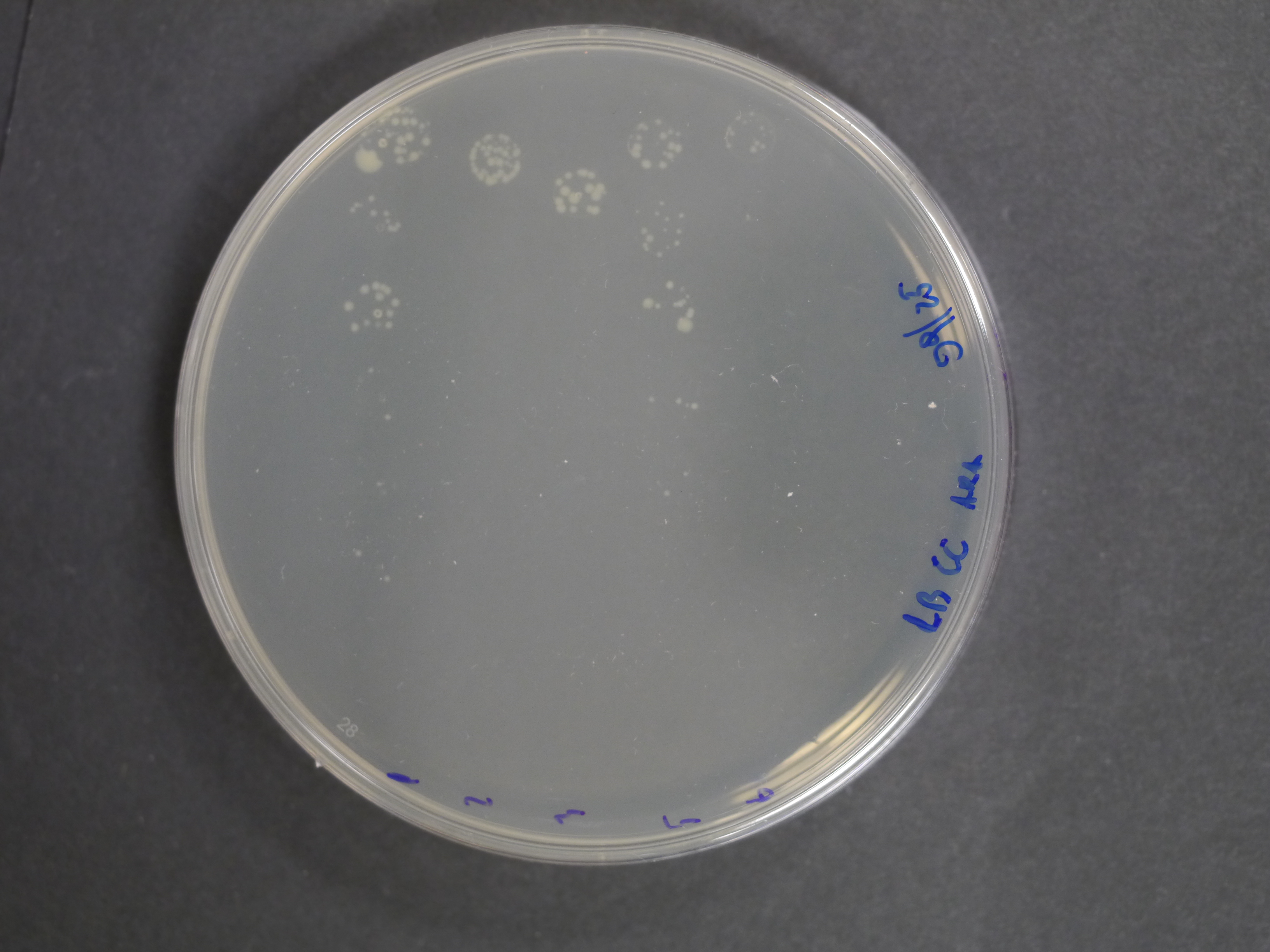

Supplement: Supplementary file 7 — Source Data [file 41467_2024_45621_MOESM7_ESM.zip › source/fig3c/P1160177.JPG]

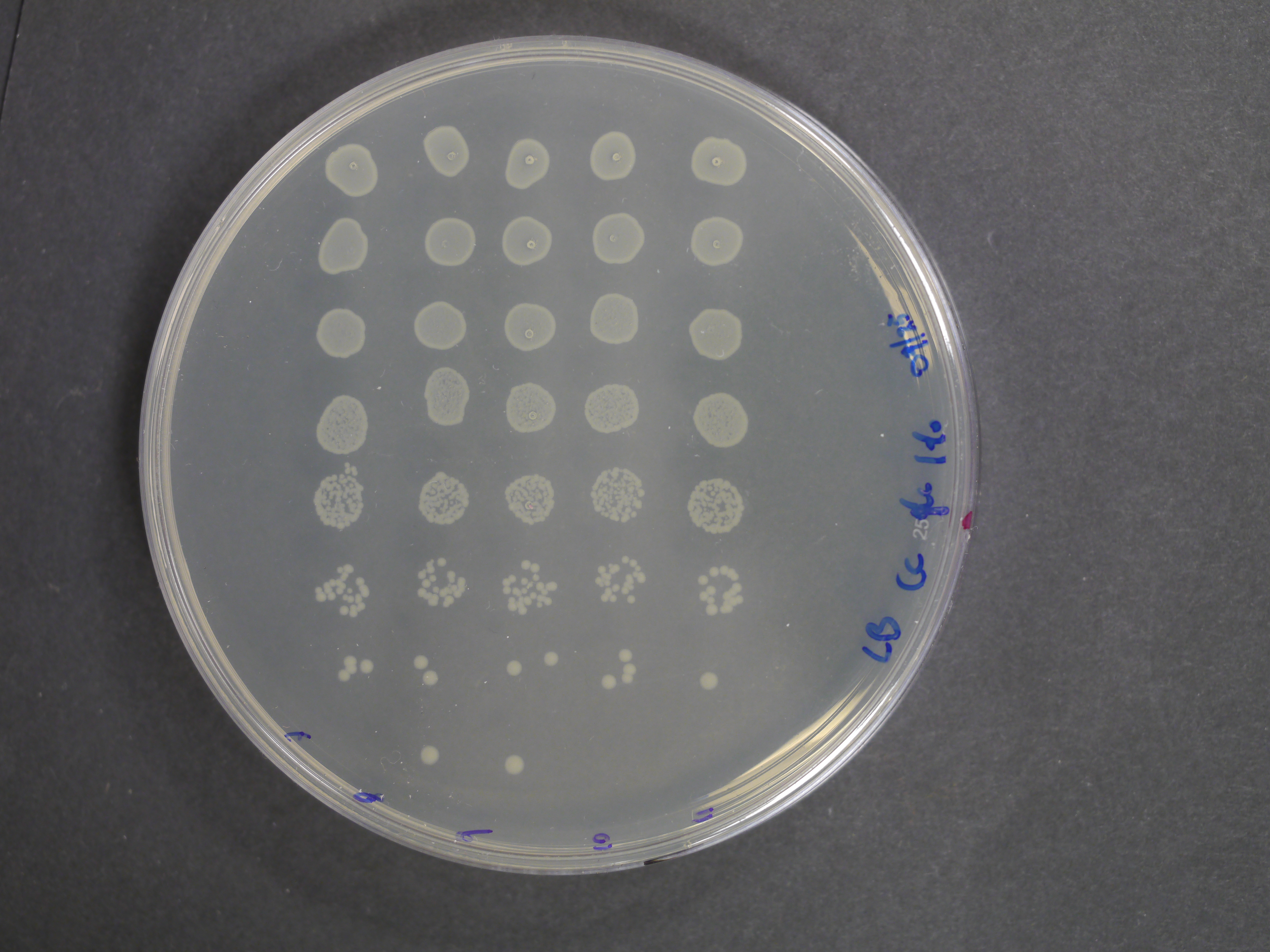

Supplement: Supplementary file 7 — Source Data [file 41467_2024_45621_MOESM7_ESM.zip › source/fig3c/P1160175.JPG]

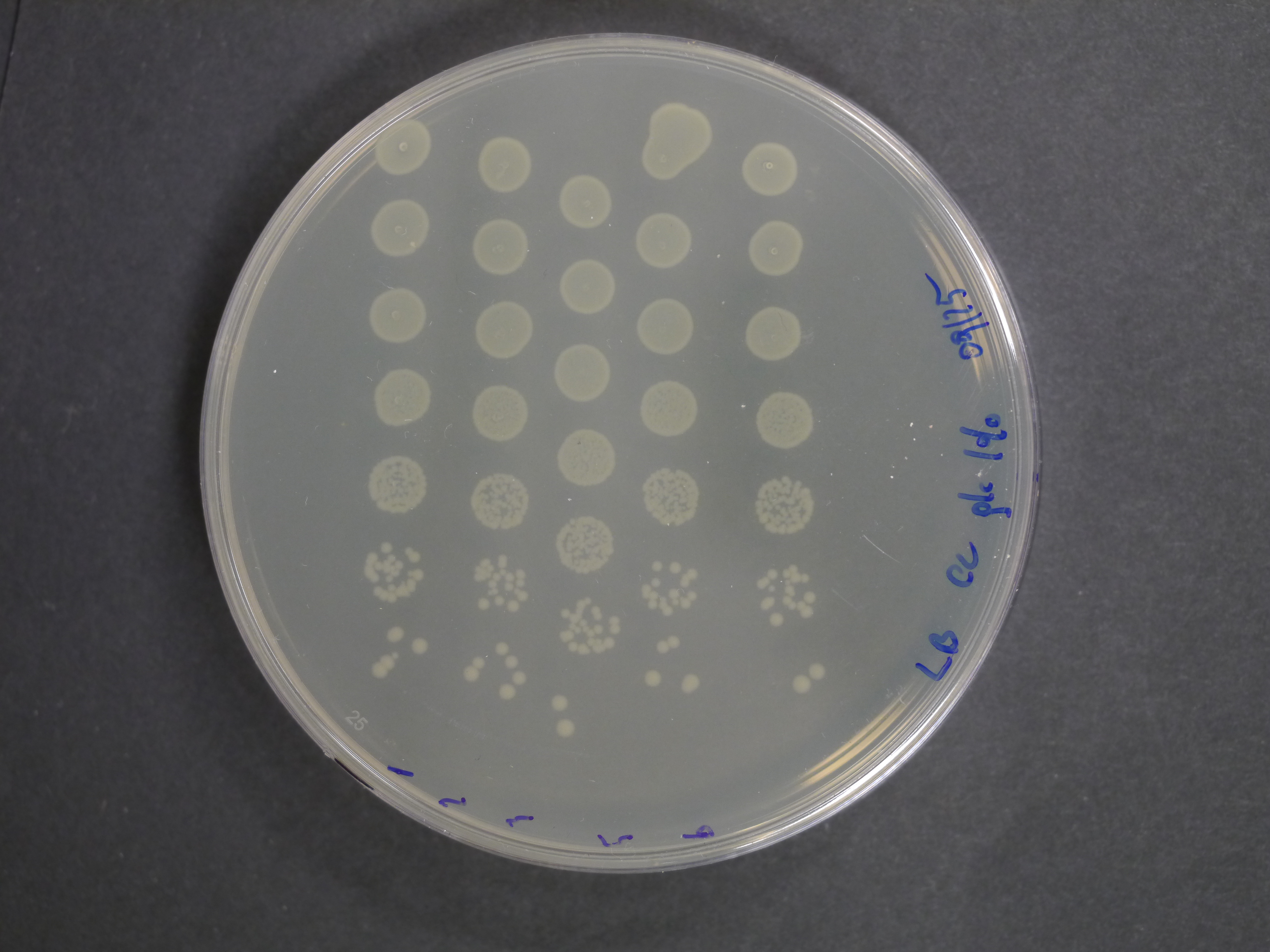

Supplement: Supplementary file 7 — Source Data [file 41467_2024_45621_MOESM7_ESM.zip › source/fig3c/P1160174.JPG]

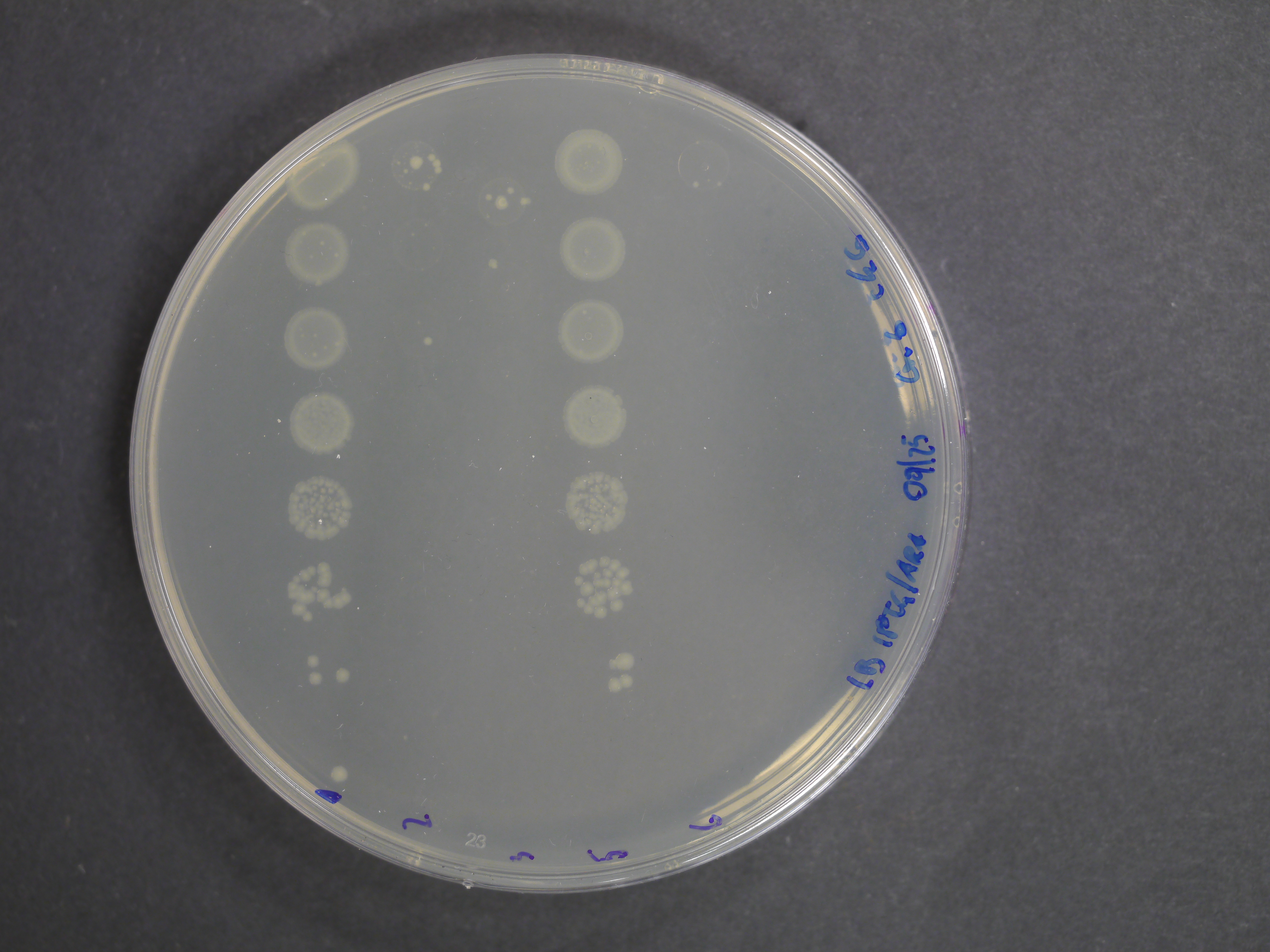

Supplement: Supplementary file 7 — Source Data [file 41467_2024_45621_MOESM7_ESM.zip › source/fig3c/P1160171.JPG]

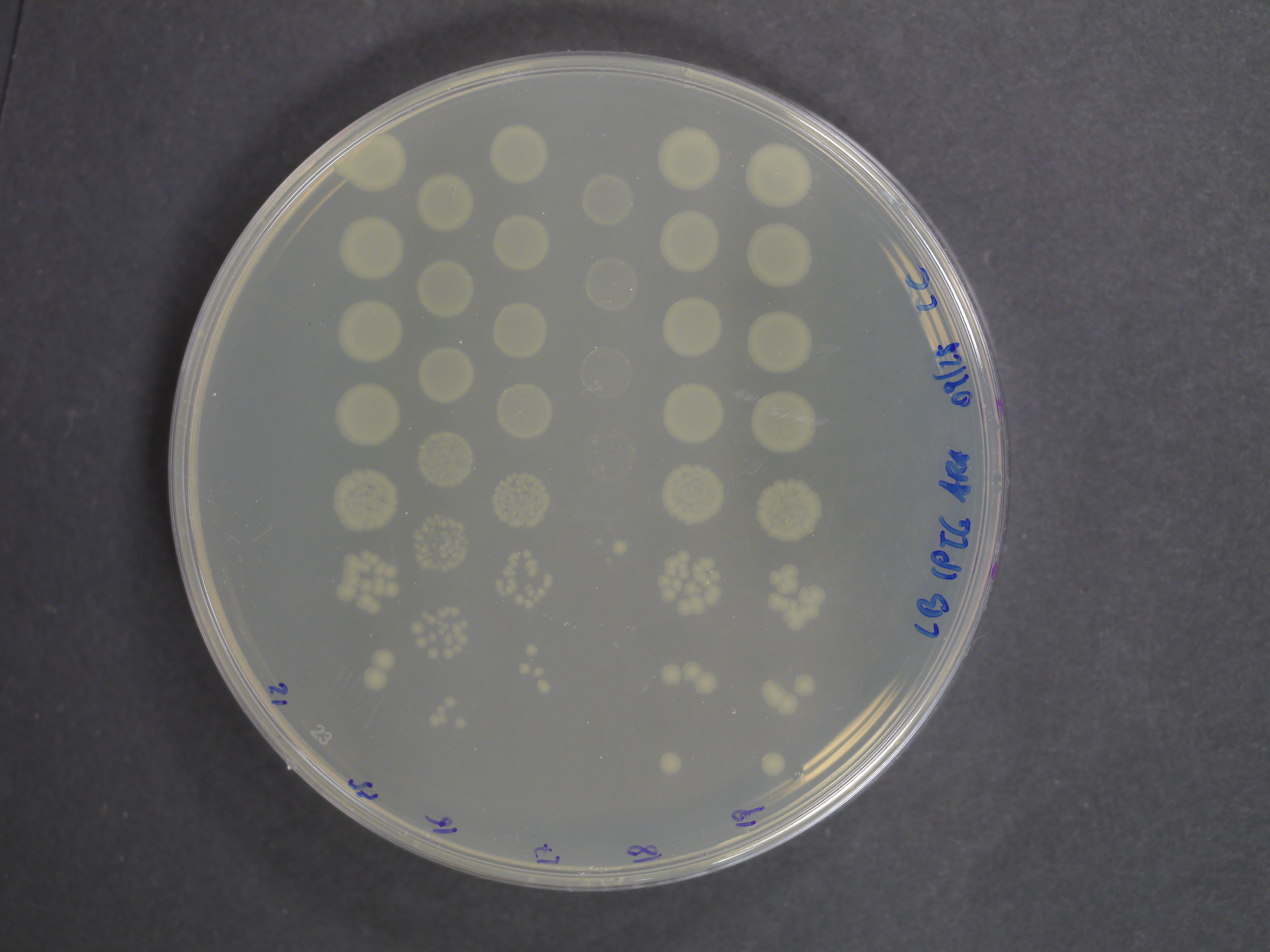

Supplement: Supplementary file 7 — Source Data [file 41467_2024_45621_MOESM7_ESM.zip › source/fig3c/P1160173.JPG]

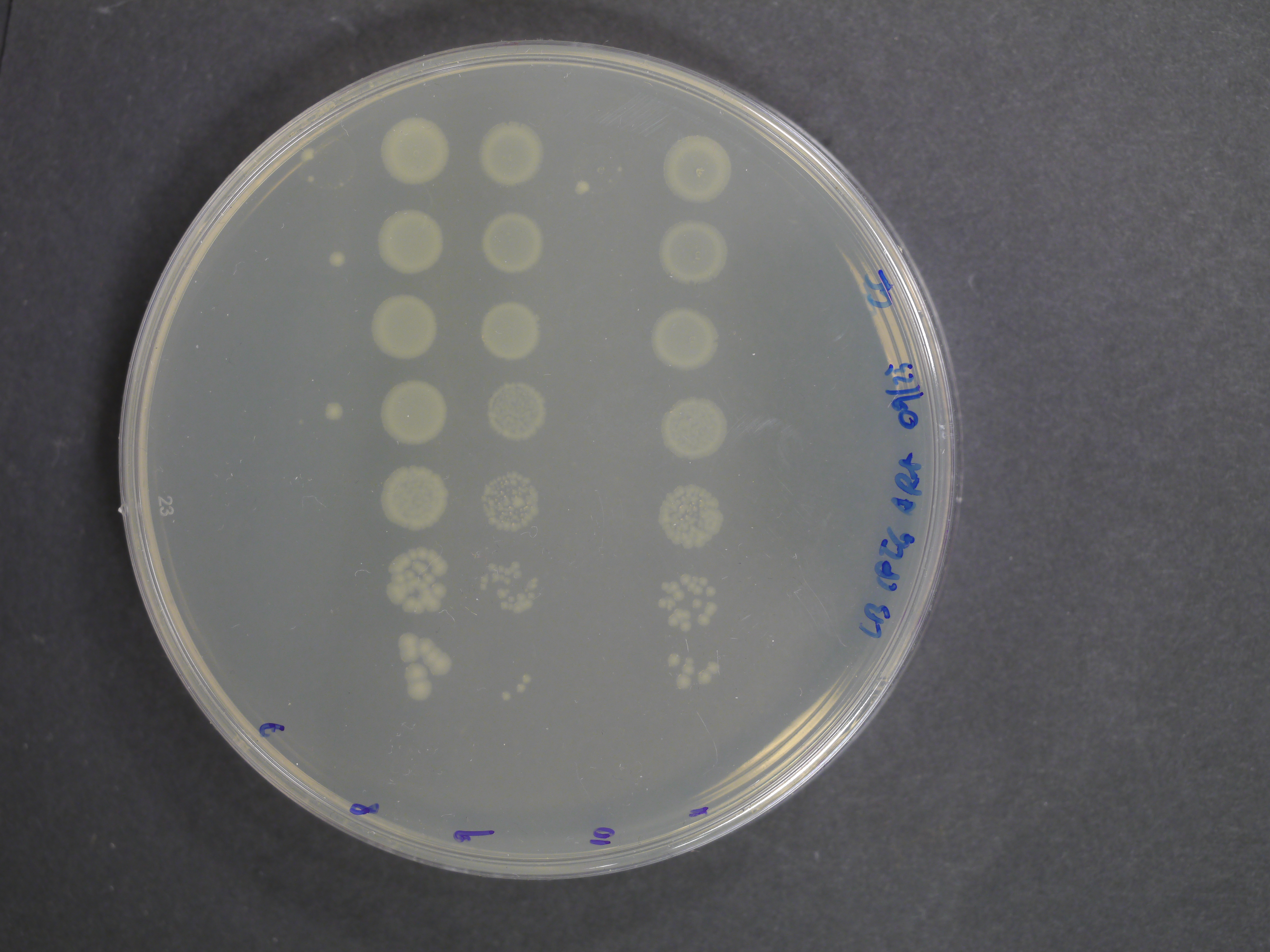

Supplement: Supplementary file 7 — Source Data [file 41467_2024_45621_MOESM7_ESM.zip › source/fig3c/P1160172.JPG]
